# Altered spectral patterns of aperiodic electroencephalography in autism

**DOI:** 10.1111/pcn.70044

**Published:** 2026-03-13

**Authors:** Yi‐Ling Chien, Ming Hsien Hsieh, Yi‐Hsuan Hsieh, Yi‐Li Tseng

**Affiliations:** ^1^ Department of Psychiatry National Taiwan University Hospital Taipei Taiwan; ^2^ Department of Electrical Engineering National Sun Yat‐sen University Kaohsiung Taiwan

**Keywords:** aperiodic component, autism, clinical correlate, E/I imbalance

## Abstract

**Aim:**

The altered ratio of neural excitation (E) and inhibition (I) has been proposed to the etiology of autism spectrum disorder (ASD). Previous studies suggested a lower E/I ratio in autistic individuals compared to non‐autistic comparisons (NACs) in specific brain regions. Whether the E/I imbalance was present in ASD in adolescence and adulthood and how the ratio correlated with clinical manifestations are unclear. This study aimed to investigate the E/I ratio in autistic adolescents and adults by using resting‐state electroencephalography (EEG) signals.

**Methods:**

The study recruited 63 autistic adolescents and adults, and 53 NAC. EEG was recorded while participants were asked to stare at the cross mark on the monitor and close their eyes for 3 min each. We used fitting oscillations and one‐over‐F function to separate periodic and aperiodic components, then fitting the curve to a linear line.

**Results:**

Autistic participants exhibited a flatter spectrum power slope and a smaller offset compared to NAC. Group differences existed in the relationship between slope and Autism Spectrum Quotient (AQ) scores, with higher AQ total scores correlated with lower aperiodic exponent in ASD. In ASD, a lower aperiodic exponent was significantly correlated with greater difficulty in mindreading, low registration, and working memory difficulty.

**Conclusions:**

The slope of the aperiodic component in ASD was flatter across frontal and posterior regions, supporting that the ASD group may have an E/I imbalance. Besides, the slope might be associated with overall autistic severity, empathy, sensory characteristics, and real‐world working memory difficulties that warrants further investigation.

Autism spectrum disorder (ASD) is a neurodevelopmental condition characterized by social communication difficulties, restricted interest, and repetitive behaviors, as well as hyper‐ or hypo‐reactivity to sensory input or unusual interests in sensory aspects of the environment.^1^ One prevailing hypothesis concerning the underlying mechanisms of ASD is the excitatory/inhibitory (E/I) imbalance hypothesis, which posits that a dysregulation between excitatory (mainly glutamatergic) and inhibitory (primarily GABAergic (γ‐aminobutyric acid)) signaling in the brain contributes to autistic condition.^2^ While causal relationships have been proposed, evidence has pointed toward both excessive excitation and excessive inhibition as potential contributors.^2^, ^3^, ^4^, ^5^, ^6^ Research has shown that infants with an older autistic sibling, who were later diagnosed with ASD themselves, exhibited heightened pupillary reactivity^7^ and increased cortical reactivity^8^, ^9^ during the first year of life. These early indicators are hypothesized to reflect an elevated E/I ratio. However, the precise mechanisms by which E/I imbalance contributes to autistic condition remain complex and are yet fully understood.^10^

Novel methods have recently been introduced as proxy measures of E/I balance through electroencephalography (EEG) recordings.^11^ This approach involves decomposing the periodic and aperiodic components, with the former reflective of traditional frequency bands and the latter indicative of irregular or nonrhythmic brain activity.^12^ The aperiodic exponent, also known as the 1/f slope, is determined by measuring the slope of the EEG power spectrum in log–log space. Depending on the sign convention used, this broadband trend may be expressed either as an exponent or as a spectral slope, with steeper spectra corresponding to larger exponents and more negative slopes. This slope has been linked to the E/I balance,^13^ where steeper slopes (i.e. higher aperiodic exponents) suggest increased inhibition relative to excitation.

Recent studies demonstrate that aperiodic brain activity provides significant insights into neural function, such as synaptic currents.^14^, ^15^ The aperiodic exponent reflects the firing statistics of underlying excitatory and inhibitory neural populations.^13^, ^16^, ^17^, ^18^ Reduced exponents, or flatter aperiodic slopes, have been associated with asynchronous, more random firing, and neural activity decoupled from canonical brain rhythms.^19^, ^20^, ^21^ Moreover, aperiodic features have been proposed to support the regulation of brain responses to environmental stimuli^22^ and outperform periodic band measures in predicting individual differences.^23^ Consistent with these findings, a recent framework suggests that aperiodic exponents may represent asynchronous excitation and ‘neural noise,’ offering a mechanistic link to oscillatory activity and behavioral outcomes.^19^, ^20^, ^21^

Emerging evidence indicates that neurodevelopmental disruptions are linked to alterations in the aperiodic exponent, with studies showing associations in conditions such as schizophrenia^24^ and attention deficit hyperactivity disorder (ADHD).^25^, ^26^, ^27^, ^28^, ^29^ Taking ADHD as an example, the aperiodic power spectral slope has been proposed as a reliable predictor of ADHD during early development^29^ Dakwar‐Kawar *et al*.^25^ reported higher aperiodic exponents in individuals with ADHD, indicative of reduced neuronal E/I balance. One‐month‐old infants with a family history of ADHD also exhibit higher aperiodic exponents compared to infants without such a history,^28^ highlighting the possibility of developmental specificity of E/I alterations^29^ To our knowledge, EEG studies on E/I alterations in ASD remain limited. A recent investigation involving preterm infants reported higher aperiodic exponents at 9 months, which correlated with increased autistic‐like behaviors later in childhood^30^ It is unclear whether the atypical aperiodic exponents can be observed in autistic adolescents or adults and their clinical correlates.

With respect to the clinical correlates of the aperiodic exponent, a flatter slope of the EEG power spectrum has been consistently observed in older individuals compared to younger ones^31^, ^32^ This flatter slope has been linked to processing speed^33^, ^34^ as well as broader domains of cognitive performance^35^, ^36^ though the association with cognitive performance may not be consistent across studies^31^ In ADHD research, the diagnosis of ADHD has been associated with a steeper spectral slope^25^, ^28^ or flatter slope^26^ Besides, the slope of the power spectrum has been reported to be associated with cognitive impairments^26^, ^27^, ^28^, ^29^ processing speed^25^ methylphenidate treatment effects^26^, ^28^ and response inhibition^27^, ^28^ Moreover, a recent study focused on temporal resolution of perception demonstrated that a flatter slope (indicative of greater neural excitation) was associated with increased sensory noise, leading to shallower psychometric curves^37^ This suggests that aperiodic EEG activity may be linked to sensory integration processes, which are often attributed to the rhythmic inhibition of neural oscillations. Whether the power spectrum slope correlates with autistic characteristics, sensory profile, and real‐world executive function is of particular interest to us, as these are neurocognitive manifestations commonly associated with the ASD phenotype.

The current study aims to investigate aperiodic component in adolescents and adults with ASD in comparison to non‐autistic comparisons (NACs). The clinical correlates of aperiodic component were examined, focusing on unique autistic characteristics, sensory profiles, empathy function, and real‐world executive functions. We hypothesized that autistic participants would exhibit a flatter slope, which would be associated with higher levels of autistic traits, sensory symptoms, executive dysfunction, and difficulties in empathy.

## Materials and Methods

### Participants and Procedure

We recruited 63 autistic individuals (aged 12.8–41.8 years, mean age 25.9 ± 6.2, male *n* = 44, 69.8%) and 53 NACs (aged 18.4–44.3 years; mean age 24.2 ± 4.4; male *n* = 28, 52.8%). Autistic individuals were referred from the outpatient clinics of the Department of Psychiatry, National Taiwan University Hospital, Taiwan. The diagnosis of ASD was made by board‐certified child psychiatrists according to the Diagnostic and Statistical Manual of Mental Disorders (5th ed.),^38^ and confirmed by the Chinese versions of the Autism Diagnostic Interview‐Revised and Autism Diagnostic Observation Scale.^39^, ^40^ The autistic participants did not have history of head injury or received surgical operation. As for psychiatric comorbidities, medical chart records revealed that 22.2% had a current or previous diagnosis of attention deficits hyperactivity disorder. Several autistic participants had a history of depressive disorder, obsessive compulsive disorder, adjustment disorder, etc (Table S1). As for medications, 20.7% of the autistic participants had been prescribed with methylphenidate, 12.7% were prescribed with serotonin reuptake inhibitors, 9.5% took aripiprazole, 6.3% took bupropion, etc. (Table S2). All medications were withheld on the day of EEG examination.

The NAC were recruited from advertisements. All the NAC participants were screened by a detailed interview conducted by trained research personnel to ensure the absence of ASD, neurological disorders, or major psychiatric conditions, including ASD. Those with any current or history of psychiatric or neurodevelopmental diagnosis were excluded.

All the participants received IQ assessment on the Wechsler Adult Intelligence Scale‐IV,^41^ reported on the clinical measures for autistic symptoms (by the Autism Spectrum Quotient, AQ), empathy (Empathy Quotient, EQ), sensory symptoms (i.e. Adult/Adolescent Sensory Profile, AASP), and real‐world executive function (i.e. Behavior Rating Inventory of Executive Function, BRIEF). In the NAC group, we used AQ < 30 and EQ > 30 as exclusion criteria to further reduce the likelihood of undetected autistic traits in the NAC group. The final NAC sample had a mean AQ score of 21.0 and a mean EQ total score of 43.5, supporting the appropriateness of their inclusion as non‐autistic controls.

The study was implemented after approval of the Research Ethics Committee at the National Taiwan University Hospital (Approval number, 201903126RINA). The study procedure is conformed to the standards of the Declaration of Helsinki. After a detailed explanation of the study objective and procedures, written informed consent was obtained from all the participants and their parents.

### Clinical measures

The AQ^42^ is a self‐report questionnaire designed to measure autistic traits in adults with normal intelligence. It comprises 50 statements that assess personal preferences, habits, and viewpoints. Each statement is rated on a four‐point scale, with ‘definitely agree’ and ‘slightly agree’ scored as “1”, and ‘slightly disagree’ and ‘disagree’ scored as “0.” In the original study, 80% of autistic individuals scored above the clinical cutoff point of 32, compared to only 2% of non‐autistic individuals exceeding this threshold.^42^ In our study, we applied a 5‐factor structure, as proposed in a psychometric study of the Chinese AQ from Taiwan,^43^ which includes the following factors, that is, Socialness, Mindreading, Patterns Preoccupation, Attention to Details, and Attention Switching.

The EQ^44^ is a self‐report questionnaire designed to assess empathy. It has demonstrated excellent internal consistency (Cronbach's alpha 0.92) and test–retest reliability (0.97). Around 81% of autistic adolescents and adults score below 30 on the EQ, compared to only 12% of non‐autistic individuals. The Mandarin Chinese version of EQ has also shown satisfactory reliability and validity (Huang HY, Gau SS, unpublished).

The AASP^45^ is a 60‐item self‐report questionnaire developed to assess sensory‐related symptoms and behaviors. It covers six sensory modalities, including taste/smell, motion, visual, tactile, activity, and auditory. Responses are rated on a 5‐point Likert scale, reflecting the frequency of occurrences: ‘never’ (0%), ‘seldom’ (25%), ‘sometimes’ (50%), ‘frequently’ (75%), and ‘always’ (100%). The scoring is based on a 4‐dimensional structure, consisting of Low Registration (15 items), Sensation Seeking (15 items), Sensory Sensitivity (15 items), and Sensation Avoiding (15 items). The psychometric properties of the Mandarin Chinese version of the AASP have been validated.^46^

The BRIEF‐A^47^ is a 75‐item self‐report rating scale developed to assess daily, real‐world executive functioning in individuals aged 18–90. All 75 items are rated in terms of frequency on a 3‐point scale: 0, ‘never’; 1, ‘sometimes’; 2, ‘often’. It comprises nine empirically derived subscales, including Inhibit, Shift, and Emotional Control subscales to assess the ability of inhibitory control to shift cognitive set and modulate emotions and behavior, as well as the Initiate, Working Memory, Plan/Organize, Organization of Materials, Task‐Monitor, and Self‐Monitor subscales to represent the ability to use working memory to initiate, plan, organize, and sustain future‐oriented problem‐solving. The BRIEF‐A has demonstrated satisfactory psychometric properties, including high inter‐rater reliability (self‐report vs informant‐report: 0.44–0.68), test–retest reliability (0.82–0.94), moderate to high internal consistency (0.73–0.90), along with good face validity and predictive validity.^48^ In this study, we used the Chinese version of the BRIEF‐A to assess executive dysfunction in everyday life.

### Experimental paradigm

Two resting‐state conditions were implemented: eyes‐closed (EC) and eyes‐open (EO). Each condition lasted for 3 min, with events occurring every 3 s. During the EO condition, a white cross was presented at the center of the monitor. The participants were instructed to close their eyes and relax before the EC condition began, to focus on the white cross for the entire duration of the EO condition, and to remain still and avoid large body movements to ensure data quality. Individuals showing an inability to maintain the required level of vigilance and attention during the initial assessment were excluded from the study. We observed that all the participants were able to maintain a stable physiological state during this brief duration.

### EEG recording and signal processing

EEG data were recorded using a Quick‐20 Dry EEG Headset (CGX, Inc., San Diego, CA, USA) with 19 channels, following the International 10–20 System, and 2 reference channels placed on the earlobes. The EEG signals were captured with CGX Acquisition Software at a sampling rate of 500 Hz and stored using the Labstreaming Layer application. Preprocessing of the EEG signals was performed using the EEGLAB toolbox.^49^ First, a high‐pass filter at 0.5 Hz was applied, and 60‐Hz line noise was removed using the CleanLine function. Bad channels were identified and removed, after which the Artifact Subspace Reconstruction (ASR) method was applied to eliminate artifacts. The removed channels were then interpolated, and the data from each channel were re‐referenced using the average signal. A zero‐phase finite‐impulse‐response (FIR) low‐pass filter at 50 Hz (filter order = 132) was subsequently applied using a forward‐reverse procedure, yielding a sufficiently steep transition band without affecting spectral characteristics below 50 Hz (Fig. S1). Finally, the Adaptive Mixture of Independent Component Analyzers (AMICA) was used to remove any remaining artifacts and reconstruct the signals.

### Aperiodic component: 1/f

**Fig. 1:**
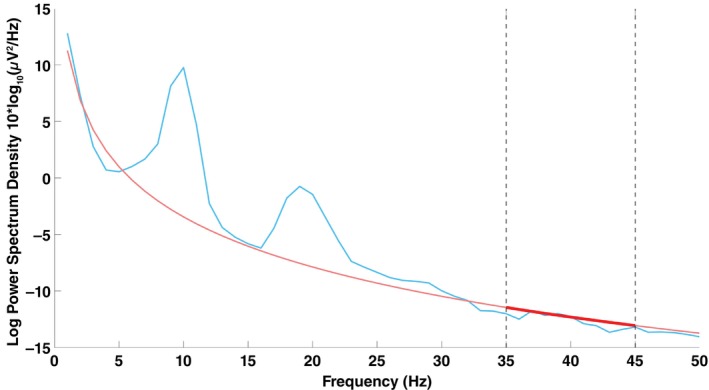
The power spectrum (blue line) with the aperiodic component separated from the original spectrum using the fitting oscillations and one‐over‐F (FOOOF) method (red line) in one non‐autistic control. A linear fit was applied to the 35–45 Hz range of the aperiodic component to minimize the influence of periodic components and line noise (red bold line).

To analyze the aperiodic component of the EEG signals, we first separated it from the periodic component. Periodic activity includes neural oscillations such as alpha waves (8–12 Hz) and beta waves (12–30 Hz), whereas aperiodic activity follows a 1/f‐like pattern in the frequency domain.^33^ The fitting oscillations and one‐over‐F (FOOOF) algorithm was employed to differentiate between the aperiodic and periodic components (Fig. 1).^11^ The aperiodic component was parameterized by an aperiodic exponent and an aperiodic offset, where the exponent characterizes the broadband 1/f spectral decay and the offset reflects broadband power in log units. A linear fit was applied to the 35–45 Hz range of the aperiodic component to minimize the influence of periodic activity and line noise. The slope of this fit, which corresponds to the negative part of the aperiodic exponent, was used as the feature representing the 1/f component. While the slope was estimated from the restricted 35–45 Hz range and the aperiodic exponent and offset were obtained from the broadband aperiodic component, these measures capture the same underlying broadband spectral characteristics and differ only marginally due to their fitting ranges. For the analysis, the brain was divided into two regions: the “frontal” region (Fz, F3, F4, F7, and F8 channels) and the “posterior” region (Pz, P3, P4, P7, P8, O1, and O2 channels). The slope was calculated for each channel within these regions.

### Statistical analysis

Statistical analyses were conducted using SAS program 9.4 (SAS Institute Inc., Cary NC, USA). The age and full‐scale IQ were compared between the ASD and NAC groups using independent *t*‐test, while sex distribution was compared using the *χ* ^2^ test. Clinical measures of autistic symptoms (i.e. the subscores and total scores of the AQ and EQ), sensory characteristics (i.e. the four subscores of the AASP), as well as the aperiodic exponent and offsets, were compared between the ASD and NAC groups by the analysis of covariance (ANCOVA), with sex and age as covariates. Because the ASD group had significantly lower full‐scale IQ than the NAC group, we examined whether group differences in aperiodic exponent (or offset) remained significant after adjusting for full‐scale IQ in addition to sex and age. The adjusted group effect remained significant after controlling for these covariates. Effect sizes were estimated by the Cohen's *d*. The effects of age and sex on the aperiodic exponent and offsets were examined using linear regression and independent *t*‐test separately.

Considering that EEG activities continue to develop until around age 25,^50^ we further divided the sample into older (age > 25) and younger (age ≤ 25) age subgroups for group comparison on the aperiodic exponent and offset by ANCOVA, with sex, age, and/or full‐scale IQ covariated.

To examine the relationship between aperiodic exponent and autistic symptoms, group‐by‐AQ interaction and group‐by‐EQ interaction were evaluated by the interaction terms in the model, controlling for sex, age, and the main effects of group and AQ/EQ. To identify the source of group interaction, Pearson correlation analyses were conducted between each aperiodic exponent (of two conditions and two brain regions) and the AQ and EQ total scores within the ASD and NAC group separately, with sex and age partial out. For significant correlations with aperiodic exponent (i.e. AQ total scores in eye‐closed condition at posterior brain region in ASD, EQ total scores in eye‐open condition at both frontal and posterior brain regions), we also examined whether similar correlations were present for the aperiodic offset.

To further explore the clinical correlates of the aperiodic exponent significantly associated with AQ total scores, we examined its correlations with mindreading deficits, sensory characteristics, and real‐world executive function. A *P*‐value of less than 0.05 was considered statistically significant.

## Results

As Table 1 shows, age and sex were not different between autistic and non‐autistic participants, yet the ASD group had a lower full‐scale IQ than the NAC group. As expected, the autistic participants had more autistic symptoms on the AQ total scores and subscores (i.e. Socialness, Mindreading, and Attention Switching Difficulty), more sensory symptoms on the AASP (with higher scores on the Low Registration, Sensory Sensitivity, and Sensory Avoiding, yet lower scores on the Sensation Seeking), and lower empathy ability on the EQ than NACs.

**Table 1:** Demographics, autistic symptoms, sensory characteristics, aperiodic components of the sample

	ASD (*n* = 63)		NAC (*n* = 53)		ASD vs NAC				
	Mean	SD	Mean	SD	*t* or *χ* ^2^	*P*			
Age	25.87	6.18	24.17	4.44	−1.72	0.0879			
Male	*n* = 44	53%	*n* = 28	70%	*χ* ^2^ = 3.538	0.06			
Full‐scale IQ	104.19	17.69	119.36	14.30	4.43	<0.0001			

					[Adj. sex, age]		[Adj. sex, age, FIQ]		
AQ total scores	102.84	12.52	82.64	11.40	7.71	<0.0001	5.13	<0.0001	
Socialness	38.76	6.28	28.86	6.95	7.1	<0.0001	4.80	<0.0001	
Mindreading	22.22	4.35	16.32	4.96	5.92	<0.0001	4.32	<0.0001	
Patterns preoccupation	12.17	3.31	11.50	2.83	0.11	0.915	0.11	0.909	
Attention to details	11.64	2.19	11.27	1.98	0.65	0.515	−0.15	0.884	
Attention switching	18.05	2.67	14.68	2.79	5.77	<0.0001	3.53	0.0007	
EQ total scores	24.28	10.88	43.50	13.97	−7.95	<0.0001	−6.48	<0.0001	
Sensory profile									
Low registration	43.07	10.01	36.63	8.76	3.95	0.0002	2.25	0.0273	
Sensation Seeking	36.45	8.61	45.63	6.78	−5.54	<0.0001	−5.03	<0.0001	
Sensory sensitivity	47.07	10.72	41.67	7.47	3.33	0.0012	2.29	0.0248	
Sensation avoiding	49.27	10.25	43.30	7.89	3.33	0.0012	4.80	<0.0001	

Aperiodic component									Cohen's *d*
Exponent									
Frontal (eye‐open)	−1.18	0.19	−1.28	0.22	2.54	0.0124	2.82	0.0059	0.501
Frontal (eye‐closed)	−1.35	0.20	−1.49	0.19	3.43	0.0008	3.18	0.0021	0.710
Posterior (eye‐open)	−1.17	0.18	−1.30	0.19	3.51	0.0007	3.11	0.0026	0.710
Posterior (eye‐closed)	−1.31	0.22	−1.42	0.21	2.65	0.0093	2.64	0.0099	0.553
Offset									
Frontal (eye‐open)	8.95	2.84	10.25	3.28	−2.1	0.0384	−1.30	0.1979	−0.423
Frontal (eye‐closed)	10.66	3.12	12.10	2.37	−2.28	0.0246	−1.65	0.1034	−0.520
Posterior (eye‐open)	9.08	2.79	10.51	2.63	−2.39	0.0187	−1.71	0.0911	−0.527
Posterior (eye‐closed)	11.03	3.14	12.60	2.76	−2.27	0.0254	−1.65	0.1036	−0.530

### Group comparison on spectrum power slope

**Fig. 2:**
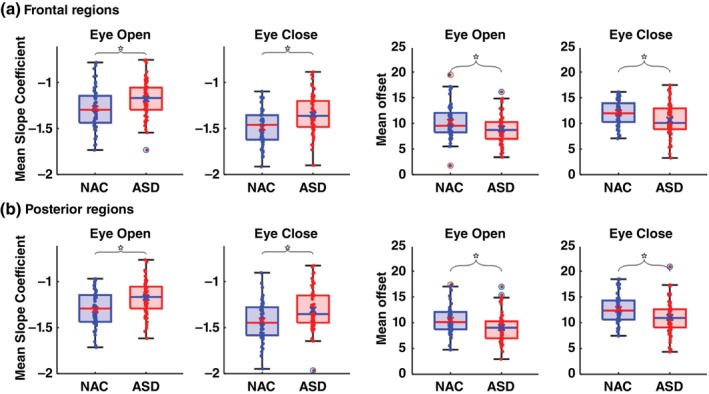
The mean slopes and offsets in the eye‐closed and eye‐open conditions at (a) frontal and (b) posterior brain regions. The horizontal lines inside the boxes represent medians, the X marks represent means, and the stars indicate significant differences.

Comparing the aperiodic component between the ASD group and NAC group in the eye‐open condition, we found that the ASD group showed a flatter spectrum power slope than the NAC group at both the frontal (ASD −1.18 ± 0.19 vs NAC −1.28 ± 0.22, *t* = 2.54, *P* = 0.0124) and posterior brain areas (ASD −1.17 ± 0.18 vs NAC −1.30 ± 0.19, *t* = 3.51, *P* = 0.0007) after adjusting for sex and age (Table 1, Fig. 2). The significant difference remained when full‐scale IQ was controlled in ANCOVA. Similarly, in the eye‐closed condition, the ASD group also showed a flatter spectrum power slope than the NAC group at both the frontal (ASD −1.35 ± 0.20 vs NAC −1.49 ± 0.19, *t* = 3.43, *P* = 0.0008) and posterior brain areas (ASD −1.31 ± 0.22 vs NAC −1.42 ± 0.21, *t* = 2.65, *P* = 0.0093) when controlling for sex, age, and/or full‐scale IQ in the ANCOVA (Table 1). The effect sizes were generally medium to high (Cohen's *d* 0.50 ~ 0.71) (Table 1).

Besides, the offset was significantly smaller in ASD than in NAC at both the frontal and posterior brain areas in both eye‐open and eye‐closed conditions (Table 1), with medium effect size (Cohen's *d* −0.42 ~ −0.53). However, if full‐scale IQ was also controlled in addition to sex and age, the significant group difference disappeared.

### Age and sex effect of aperiodic component in ASD and NAC

We examined age effect on each index of the aperiodic component in ASD and NAC separately. We found significant correlation with age only at the frontal brain area in the eye‐close condition but not the frontal regions or eye‐open condition. Though, significant group‐by‐age interaction was found in the anterior eye‐close (*F* = 5.88, *P* = 0.0169) and posterior eye‐open conditions (*F* = 4.11, *P* = 0.045) (Fig. S2).

We therefore divided the sample by age 25 and found significant ASD‐NAC differences remained in most indices in the older subgroup (age > 25) but not in the younger subgroup (age ≤ 25) (Table S3).

As for sex effect, there was no sex difference in NAC for 1/f in each area or condition. Whether, the power spectrum slope was generally more flatten in autistic males than in autistic females, but only the posterior area in the eye‐close condition showed statistical significance (*t* = −2.16, *P* = 0.0346). The sex‐by‐group interaction was not significant.

There was no sex difference of offset in each group. Yet, a significant age effect was found in the ASD group but not in the NAC group, showing that older autistic participants had a smaller offset (Fig. S3).

### The relationship between aperiodic component and autistic symptoms

Before exploring the clinical correlates of the aperiodic component, we examined ASD‐NAC group differences in the relationship between the slope and the autistic symptoms measured by the AQ and EQ total scores in all the participants. We found a significant interaction between group and the AQ total scores across frontal (*t* = 2.6, *P* = 0.011) and posterior brain regions (*t* = 2.56, *P* = 0.012) in the eye‐closed condition (Table S4). We also found a significant interaction between group and the EQ total scores at the frontal area in the eye‐open condition (*t* = 2.84, *P* = 0.0055).

**Fig. 3:**
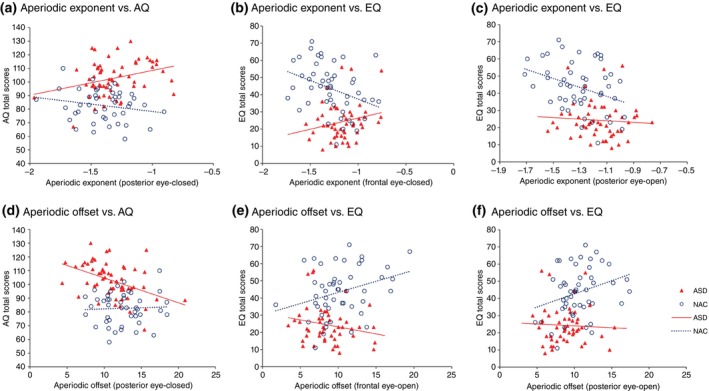
Relationship between aperiodic exponent and autistic characteristics on the AQ and EQ total scores. (a) Aperiodic exponent versus AQ, (b, c) Aperiodic exponent versus EQ, (d) Aperiodic offset versus AQ, (e, f) Aperiodic offset versus EQ.

Separating the ASD group from the NAC group, we found a significant correlation between the exponent (eye‐closed condition in posterior brain, *r* = 0.281, *P* = 0.034) and the AQ total scores in ASD only (Fig. 3a), and a significant correlation between exponent (eye‐open condition in both frontal [*r* = −0.365, *P* = 0.011] and posterior brain [*r* = −0.330, *P* = 0.022]) and the EQ total scores in NAC only (Fig. 3b,c).

We specifically tested these correlations with offset and found that the offset of posterior brain of the eye‐close condition was significantly negatively correlated with AQ total scores in ASD (*r* = −0.300, *P* = 0.043) (Fig. 3d). While in the NAC group, positive correlations were found between the offset in the eye‐open condition (frontal area, *r* = 0.370, *P* = 0.017; posterior *r* = 0.321, *P* = 0.041) and the EQ total scores (Fig. 3e,f).

### Clinical correlates of aperiodic component in ASD

**Fig. 4:**
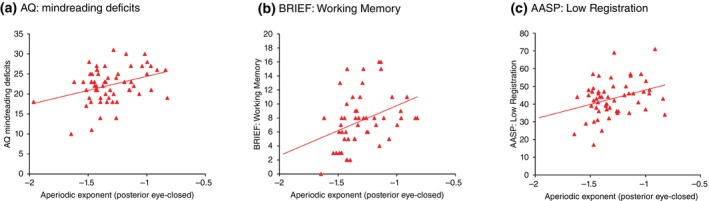
Relationship between aperiodic exponent and clinical correlates. (a) AQ: Mindreading deficits. (b) BRIEF: Working memory, (c) AASP: Low registration.

Given the significant lower aperiodic exponent at posterior brain in the eye‐close condition and its association with AQ total scores in ASD, we tested the clinical correlates of this index among the phenotypes of ASD, including mindreading deficits (Fig. 4a), real‐world executive functions (Fig. 4b), and sensory characteristics (Fig. 4c) in the ASD group.

We examined the correlation of this index for each AQ subscore and found that only the mindreading subscale was significantly correlated with the exponent at posterior brain (eye‐open: *r* = 0.287, *P* = 0.028; eye‐closed: *r* = 0.343, *P* = 0.008), suggesting that the correlation with overall autistic severity was mainly driven by mindreading difficulty (Fig. 4a).

We next examined the correlation of this index with the four subscores of the AASP. We found that the index was significantly positively correlated with Low Registration (*r* = 0.354, *P* = 0.016) but not with the other three AASP subscores (Fig. 4b), showing that the lower aperiodic exponent was related to the higher scores on the Low Registration subscore.

As for the relationship with the real‐world executive functions, we examined the correlation between this index and the BRIEF‐A subscales. Among the BRIEF‐A subscales, only the working memory subscale (*r* = 0.299, *P* = 0.037) was significantly correlated with the spectrum exponent (Fig. 4c). The correlations of the other subscale were not statistically significant.

## Discussion

This study yielded four major findings. First, the autistic participants exhibited a lower aperiodic exponent and a smaller offset compared to NACs, with medium effect sizes (exponent: 0.5 ~ 0.7, offset: 0.4 ~ 0.5). Second, autistic males showed a flatter slope in the posterior brain regions than autistic females, while older autistic participants had a smaller offset than their younger counterparts. Third, group differences existed in the relationship between the slope and AQ (both frontal and posterior brains in eye‐closed condition) or EQ total scores (frontal region in eye‐open condition), with higher AQ total scores correlated with lower aperiodic exponent in the ASD group and lower EQ total scores correlated with lower aperiodic exponent in NAC. Fourth, within the autistic participants, a flatter slope was significantly correlated with greater difficulty in mindreading, sensory processing (low registration), and working memory difficulty. Notably, the power spectrum slope was not correlated with empathy in the autistic participants.

### ASD showed flatter spectra than non‐ASD comparison

Our findings showed that the autistic participants exhibited a significantly flatter spectrum power slope than NACs across both frontal and posterior brains, in both eye‐closed and eye‐open conditions, with a medium effect size. The slope of the aperiodic component is considered a proxy for the E/I balance,^13^ where a steeper slope (larger aperiodic exponent) reflects greater neural inhibition relative to excitation, while flatter slopes (i.e. smaller exponents) indicate a higher excitatory‐to‐inhibitory activity ratio.^13^ In this sense, the flatter slope observed in the autistic participants implied a greater ratio of excitatory‐to‐inhibitory activity in this group across anterior and posterior brain regions in both eye‐closed and eye‐open conditions in autistic adolescents and adults, in support with the hypothesis of E/I imbalance.

Compared to the higher aperiodic exponents observed in the early developmental stage of ADHD relative to non‐ADHD controls^25^, ^28^ the smaller aperiodic exponents found in autistic individuals may support the developmental specificity of E/I alterations proposed by Karalunas *et al*.^29^ Of note, such comparison needs to be interpreted in the context of the natural age‐related decline in the aperiodic exponent. For instance, a prior study reported that higher aperiodic exponents at 9 months were correlated with increased autistic‐like behaviors later in childhood^30^ Further longitudinal studies extending beyond adolescence are essential to map the developmental trajectory of E/I alterations in autism and other neurodevelopmental disorders^51^ providing insights into the evolving nature of aperiodic EEG components throughout brain development.

### Age effect and Sex difference on power spectrum slope

When stratifying participants by age using a 25‐year cutoff, group differences in the aperiodic exponent and offset were observed exclusively in individuals aged 25 and older; no detectable ASD‐NAC differences were present in the younger subgroup (<25 years). Given that EEG maturation continues into early adulthood, this pattern suggests that atypicalities in aperiodic neural activity may emerge or become more apparent later in development. Such findings potentially reflect age‐related divergence in cortical maturation processes, specifically related to excitation–inhibition dynamics. While the relatively small sample size within each age subgroup necessitates a cautious interpretation, these results nonetheless raise the possibility that age acts as a moderating factor in the expression of aperiodic EEG alterations in autism. Previous studies have revealed that the aperiodic exponent was dependent on age, with flatter spectra consistently reported in older individuals compared to younger ones.^20^, ^31^, ^32^, ^52^, ^53^ Using linear regression to examine age effect, we found significant age effect only in the frontal brain region during the eye‐closed condition, that revealed older age was related to a flatter slope. This implies that age‐related changes in the aperiodic exponent may only emerge within a specific age period or over a broader age range.

The novel finding that autistic males exhibited a more pronounced flattening of the power spectrum slope in the posterior brain region during the eye‐closed condition compared to autistic females suggested a potential sex difference in E/I imbalance. This sex difference was not observed in the non‐autistic participants. Specifically, E/I imbalance was found to be greater in autistic males than in autistic females, but such difference was not detected in the NAC group. These findings may reflect established sex differences in the autistic brain^54^ as well as differences in symptom characteristics and behavioral responses^55^ The role of E/I imbalance in the posterior brain region and how it may modulate the clinical presentation of autistic symptoms across sexes warrants further investigation.

### Clinical correlates of aperiodic exponent

The flatter spectra have been associated with cognitive decline (Tran *et al*., 2020) and reduced processing speed^33^, ^34^ as well as broader domains of cognitive performances such as working memory, higher‐order visuospatial and verbal skills^35^ and controlled process but not automatic process^36^ Also, flatter spectra may mediate cross‐sectional associations between age and cognitive function^21^ In the present study, we found that lower aperiodic exponent in the eye‐open condition among NAC were associated with lower empathy function, expanding the understanding of the clinical implications of the aperiodic exponent in the general population. While clinical correlates of the aperiodic exponent have been researched in ADHD, showing associations with cognitive impairments,^26^, ^27^, ^28^, ^29^ processing speed,^25^ stimulant treatment effects,^26^, ^28^ and response inhibition,^27^, ^28^ similar research in autism remains limited. In this study, we found that lower aperiodic exponent in the posterior brain during eye‐closed condition in the autistic participants were associated with greater overall autistic severity and mindreading difficulty. Additionally, lower aperiodic exponent in the eye‐open condition were linked to lower empathy function in NAC. These findings suggested that resting‐state E/I balance might be related to empathy function and autistic manifestations. Furthermore, lower aperiodic exponent observed in the posterior brain during eye‐closed condition in the autistic participants were not only linked to overall autistic severity and mindreading difficulty but also with increased sensory hypo‐responsiveness, as indicated by Low Registration of the AASP, and with working memory difficulties. The relationship between flatter spectra and working memory has not been reported and warrants further investigation.

Taken together, the autistic participants not only exhibited a flatter aperiodic exponent; the flatter aperiodic exponent was also associated with core autistic symptoms, overall autistic severity, and distinct sensory characteristics. These results suggest that a flattened aperiodic exponent may serve as a potential neurophysiological marker for ASD diagnosis and severity. Future studies may explore whether cortical overexcitation of the posterior brain during eye‐closed condition reflects intrinsic cortical dysregulation that impacts holistic functioning involving sensory, social, and working memory domains. Additionally, given the flatter slope was observed in resting state, whether these findings are incorporated into default mode network dysfunction measured by the resting‐state functional MRI^56^, ^57^ awaits further investigation.

The finding of a smaller aperiodic offset in autistic individuals is intriguing. This smaller offset is correlated with older age in autism and was correlated with overall autistic severity in autism as well as lower EQ in NAC. Aperiodic offset is defined as power at 2.5 Hz, and it is demonstrated to increase with age in the first year of life^51^ It has been suggested that aperiodic offset represents broadband neuronal firing,^58^, ^59^ and early increases in aperiodic offset may align with established increases in neuronal number, gray matter volume, and synaptic density during the first year.^60^, ^61^, ^62^ The clinical implication of aperiodic offset in adolescence and adulthood remains unclear, particularly as synaptic pruning may stabilize while certain parts of gray matter may continue growing until twenties.^63^ However, given the cumulative data linking altered gray matter volume in specific brain regions in autism and synaptic neurotransmission serving as a critical mechanism in the etiology of autism, further research to elucidate its clinical relevance is of significant value.

Several limitations of this study need to be acknowledged. First, all the autistic participants had normal intelligence, which ensures the lower heterogeneity of the sample but limits the generalizability of the findings to the entire autistic spectrum. The ASD group exhibited lower intelligence than the NAC group. Although the group difference in aperiodic exponent remained significant when full‐scale IQ covariated, the IQ effect on the aperiodic exponent needs further evaluation, particularly in lower intelligence population. Besides, our sample included fewer female participants, which may have reduced the statistical power for gender‐specific analyses. Future research should validate these findings in more diverse autistic populations, including individuals with intellectual disabilities and an equal representation of female participants. Second, clinical correlates of this study were measured by self‐report questionnaires, while normal intelligence of the sample ensures the comprehension ability of the participants and supports the validity of self‐reports. Third, the NAC participants were screened through a detailed interview conducted by trained research personnel to ensure the absence of ASD, neurological disorders, or major psychiatric conditions, as well as AQ < 30 and EQ > 30 to exclude undetected autistic traits in the NAC group. However, a formal diagnostic assessment (e.g. the Structured Clinical Interview for DSM Disorders or the Kiddie Schedule for Affective Disorders and Schizophrenia) was not conducted for the NAC group, that should be considered in future studies. Besides, the lack of family history information in both ASD and NAC should also be addressed in future studies. Nevertheless, as the first report in autistic adolescents and adults, this study explored clinical correlates of 1/f in autism through a comprehensive assessment using multiple questionnaires. The explorative findings provide novel evidence as a foundation for further research.

## Conclusion

We found a flatter aperiodic exponent across the brain regions in autistic individuals under both eye‐closed and eye‐open conditions, implying an E/I imbalance in autistic adolescents and adults. This flatter slope was associated with overall autistic traits and specific core symptom—mindreading deficits, as well as low registration and working memory deficits, indicating that the aperiodic exponent may serve as a neurophysiological marker for autistic traits and associated cognitive and social deficits. Moreover, the link between the aperiodic exponent and clinical features of autism suggests that further exploration into the neurochemistry of specific brain regions—particularly the posterior regions—could provide deeper insights into the underlying mechanisms of autism. This might open avenues for developing targeted interventions aiming at restoring E/I balance and addressing cognitive and behavioral difficulties in autism. Overall, this study advances our understanding of the neurophysiological underpinnings of autism and lays the groundwork for future research aimed at refining clinical assessments and developing neurobiologically informed interventions.

## Author contributions

Y.L.C and Y.L.T. contributed to the study concept and design and performed testing and data collection. Y.L.T. performed analyses of aperiodic electroencephalography. Y.L.C. performed data analysis. Y.L.C. drafted the manuscript, and Y.L.T. provided critical revisions. All authors approved the final version of the paper for submission.

## Disclosure statement

The authors declare no conflicts of interest.

## Supporting information


**Table S1.** History of psychiatric comorbidities in autistic participants according to medical chart records.
**Table S2.** Current or history of medications in autistic participants (all medications withheld on the day of electroencephalography [EEG]).
**Table S3.** Group differences stratified by age 25, (A) ASD versus NAC in older subgroup (age > 25), (B) ASD versus NAC in younger subgroup (age ≤ 25).
**Table S4.** Group‐by‐AQ/EQ interactions and Pearson's correlations between aperiodic components and AQ/EQ in ASD and in NAC.
**Figure S1.** (A) Magnitude response of the zero‐phase FIR low‐pass filter (50 Hz cutoff, order = 132) showing a flat passband below 50 Hz. (B) Representative power spectrum displayed up to 70 Hz, illustrating preserved spectral features in the 35–45 Hz range.
**Figure S2.** Age‐related changes of aperiodic exponent.
**Figure S3.** Age‐related changes of aperiodic offset.

## Data Availability

The data that support the findings of this study are available on request from the corresponding author. The data are not publicly available due to privacy or ethical restrictions.
