# Supplementary material for: Altered spectral patterns of aperiodic electroencephalography in autism
Source: Psychiatry Clin Neurosci. 2026 Mar 13;80(5):423–31. doi: 10.1111/pcn.70044 (PMC13139815; doi:10.1111/pcn.70044)

**Supplementary Table S1.** History of psychiatric comorbidities in autistic participants according to medical chart records

|  | n | % |
| --- | --- | --- |
| Attention deficits hyperactivity disorder | 14 | 22.2% |
| Adjustment disorder with depressed mood | 3 | 4.8% |
| Adjustment disorder with anxiety mood | 2 | 3.2% |
| Fibromyalgia | 1 | 1.6% |
| Emotional disorder | 2 | 3.2% |
| Generalized anxiety disorder | 1 | 1.6% |
| Depressive disorder | 3 | 4.8% |
| Obsessive compulsive disorder | 2 | 3.2% |
| Social phobia | 1 | 1.6% |
| Tic disorder | 1 | 1.6% |

**Supplementary Table S2.** Current or history of medications in autistic participants (all medications withheld on the day of EEG)

|  | n | % |
| --- | --- | --- |
| Aripiprazole (Abilify) | 6 | 9.5% |
| Methylphenidate (Concerta) | 2 | 3.2% |
| Methylphenidate (Ritalin) | 11 | 17.5% |
| Risperidone | 2 | 3.2% |
| Serotonin norepinephrine reuptake inhibitors | 1 | 1.6% |
| Serotonin reuptake inhibitors | 8 | 12.7% |
| Bupropion (Wellbutrin) | 4 | 6.3% |

**Supplementary Table S3.** Group differences stratified by age 25, (A) ASD vs. NAC in older subgroup (age > 25), (B) ASD vs. NAC in younger subgroup (age < 25)

1. ASD vs. NAC in older subgroup (age > 25)

| Age>25 | ASD  (n=28) | | NAC  (n=33) | | ASD:NAC  (Adj. sex, age) | | ASD:NAC  (Adj. sex, age, FIQ) | | Kruskal-Wallis | |
| --- | --- | --- | --- | --- | --- | --- | --- | --- | --- | --- |
|  | mean | SD | mean | SD | t | p | t | p |  | |
| ***Aperiodic Exponent*** |  |  |  |  |  |  |  |  |  | |
| Frontal (eye-open) | -1.17 | 0.17 | -1.27 | 0.26 | 1.84 | 0.0718 | 1.79 | 0.0812 | 3.757 | 0.0526 |
| Frontal (eye-closed) | -1.29 | 0.18 | -1.55 | 0.20 | 5.22 | <.0001 | 4.5 | <.0001 | 14.747 | 0.0001 |
| Posterior (eye-open) | -1.14 | 0.16 | -1.37 | 0.19 | 4.4 | <.0001 | 4.17 | 0.0002 | 13.924 | 0.0002 |
| Posterior (eye-closed) | -1.29 | 0.21 | -1.50 | 0.17 | 3.68 | 0.0006 | 3.24 | 0.0026 | 10.572 | 0.0011 |
| ***Offset*** |  |  |  |  |  |  |  |  |  |  |
| Frontal (eye-open) | 8.53 | 2.64 | 9.97 | 4.26 | -1.41 | 0.1661 | -0.63 | 0.531 | 1.971 | 0.1604 |
| Frontal (eye-closed) | 9.52 | 2.62 | 12.19 | 2.64 | -3.92 | 0.0003 | -3.45 | 0.0015 | 9.650 | 0.0019 |
| Posterior (eye-open) | 8.51 | 2.52 | 10.88 | 3.22 | -2.88 | 0.0058 | -2.3 | 0.0271 | 7.731 | 0.0054 |
| Posterior (eye-closed) | 10.46 | 2.98 | 12.62 | 2.75 | -2.72 | 0.0088 | -2.31 | 0.027 | 5.168 | 0.023 |

(B) ASD vs. NAC in younger subgroup (age < 25)

| Age<25 | ASD  (n=35) | | NAC  (n=20) | | ASD:NAC  (Adj. sex, age) | | ASD:NAC  (Adj. sex, age, FIQ) | | Kruskal-Wallis | |
| --- | --- | --- | --- | --- | --- | --- | --- | --- | --- | --- |
|  | mean | SD | mean | SD | t | p | t | p |  | |
| ***Aperiodic Exponent*** |  |  |  |  |  |  |  |  |  | |
| Frontal (eye-open) | -1.20 | 0.23 | -1.29 | 0.21 | 1.89 | 0.063 | 2.27 | 0.029 | 2.465 | 0.116 |
| Frontal (eye-closed) | -1.42 | 0.21 | -1.45 | 0.17 | 0.86 | 0.391 | 1.26 | 0.215 | 0.151 | 0.697 |
| Posterior (eye-open) | -1.21 | 0.20 | -1.27 | 0.18 | 0.80 | 0.427 | 0.52 | 0.603 | 1.621 | 0.203 |
| Posterior (eye-closed) | -1.32 | 0.23 | -1.38 | 0.22 | 0.93 | 0.354 | 0.93 | 0.360 | 1.096 | 0.295 |
| ***Offset*** |  |  |  |  |  |  |  |  |  |  |
| Frontal (eye-open) | 9.47 | 3.04 | 10.40 | 2.65 | -2.12 | 0.039 | -1.92 | 0.062 | 1.156 | 0.282 |
| Frontal (eye-closed) | 12.08 | 3.14 | 12.05 | 2.25 | -1.01 | 0.319 | -1.11 | 0.272 | 0.034 | 0.854 |
| Posterior (eye-open) | 9.78 | 2.99 | 10.30 | 2.27 | -1.18 | 0.244 | -0.71 | 0.481 | 1.514 | 0.219 |
| Posterior (eye-closed) | 11.74 | 3.23 | 12.58 | 2.81 | -2.21 | 0.031 | -1.83 | 0.074 | 1.621 | 0.203 |

**Supplementary Table S4.** Group-by-AQ/EQ interactions and Pearson’s correlations between aperiodic components and AQ/EQ in ASD and in NAC

|  | Group*AQ interaction | | **ASD** | | **NAC** | |
| --- | --- | --- | --- | --- | --- | --- |
| **Aperiodic exponent** | *t* | *p* | *r* | *p* | *r* | *p* |
| Frontal eye-open | 0.720 | 0.475 | 0.073 | 0.591 | -0.054 | 0.735 |
| Frontal eye-closed | 2.600 | 0.011 | 0.193 | 0.150 | -0.255 | 0.103 |
| Posterior eye-open | 1.560 | 0.121 | 0.157 | 0.244 | -0.097 | 0.543 |
| Posterior eye-closed | 2.560 | 0.012 | 0.281 | 0.034 | -0.197 | 0.211 |
|  | Group*EQ interaction | | **ASD** | | **NAC** | |
| **Aperiodic offset** | *t* | *p* | *r* | *p* | *r* | *p* |
| Frontal eye-open | 2.840 | 0.006 | 0.263 | 0.072 | -0.365 | 0.011 |
| Frontal eye-closed | 0.430 | 0.669 | -0.116 | 0.432 | -0.176 | 0.231 |
| Posterior eye-open | 1.190 | 0.238 | -0.103 | 0.487 | -0.330 | 0.022 |
| Posterior eye-closed | -0.150 | 0.880 | -0.177 | 0.228 | -0.162 | 0.272 |

**Note.** Sex and age were controlled in the analyses

**Supplementary Figure S1.** (A) Magnitude response of the zero-phase FIR low-pass filter (50 Hz cutoff, order = 132) showing a flat passband below 50 Hz. (B) Representative power spectrum displayed up to 70 Hz, illustrating preserved spectral features in the 35–45 Hz range.


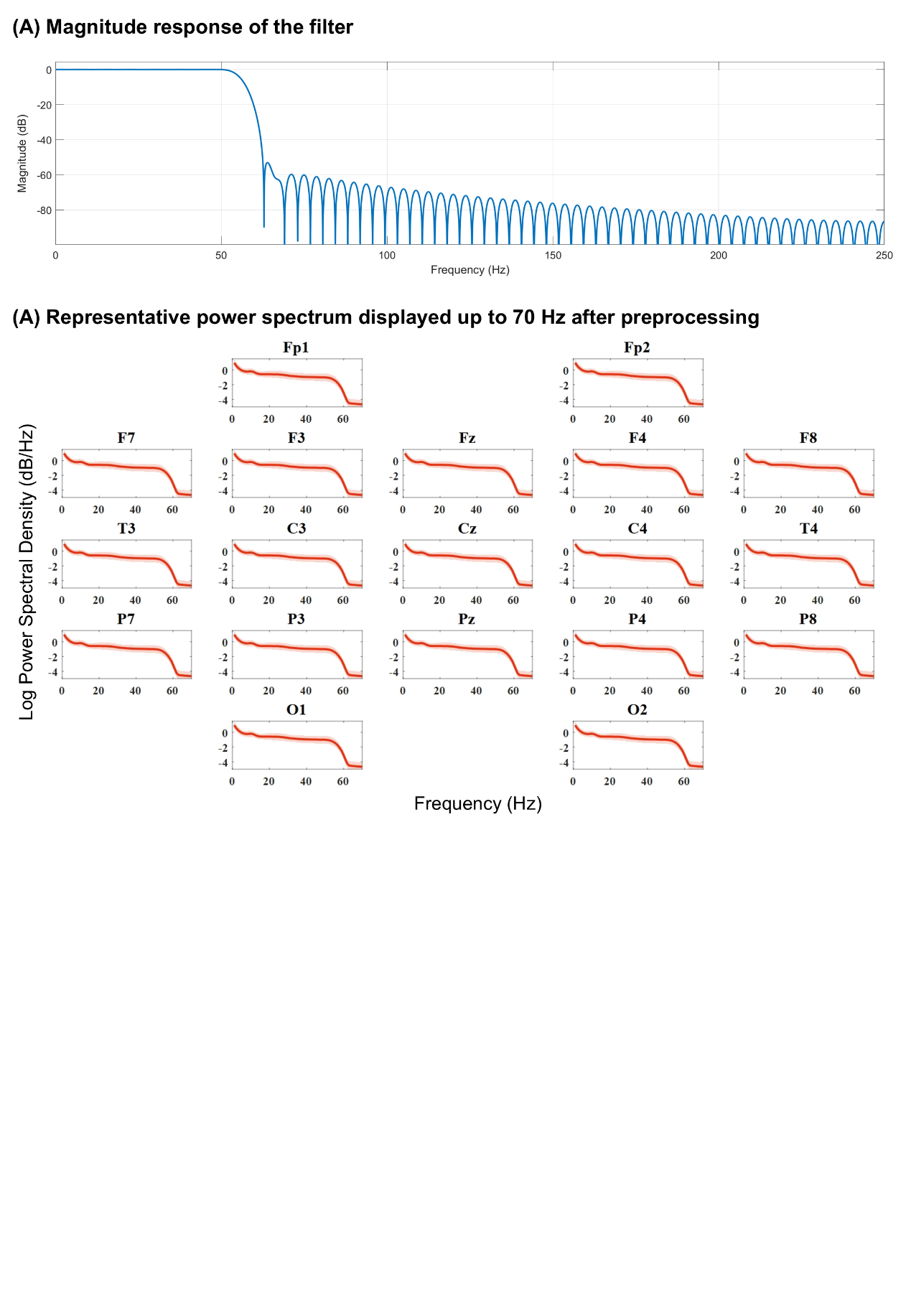


**Supplementary Figure S2.** Age-related changes of aperiodic exponent


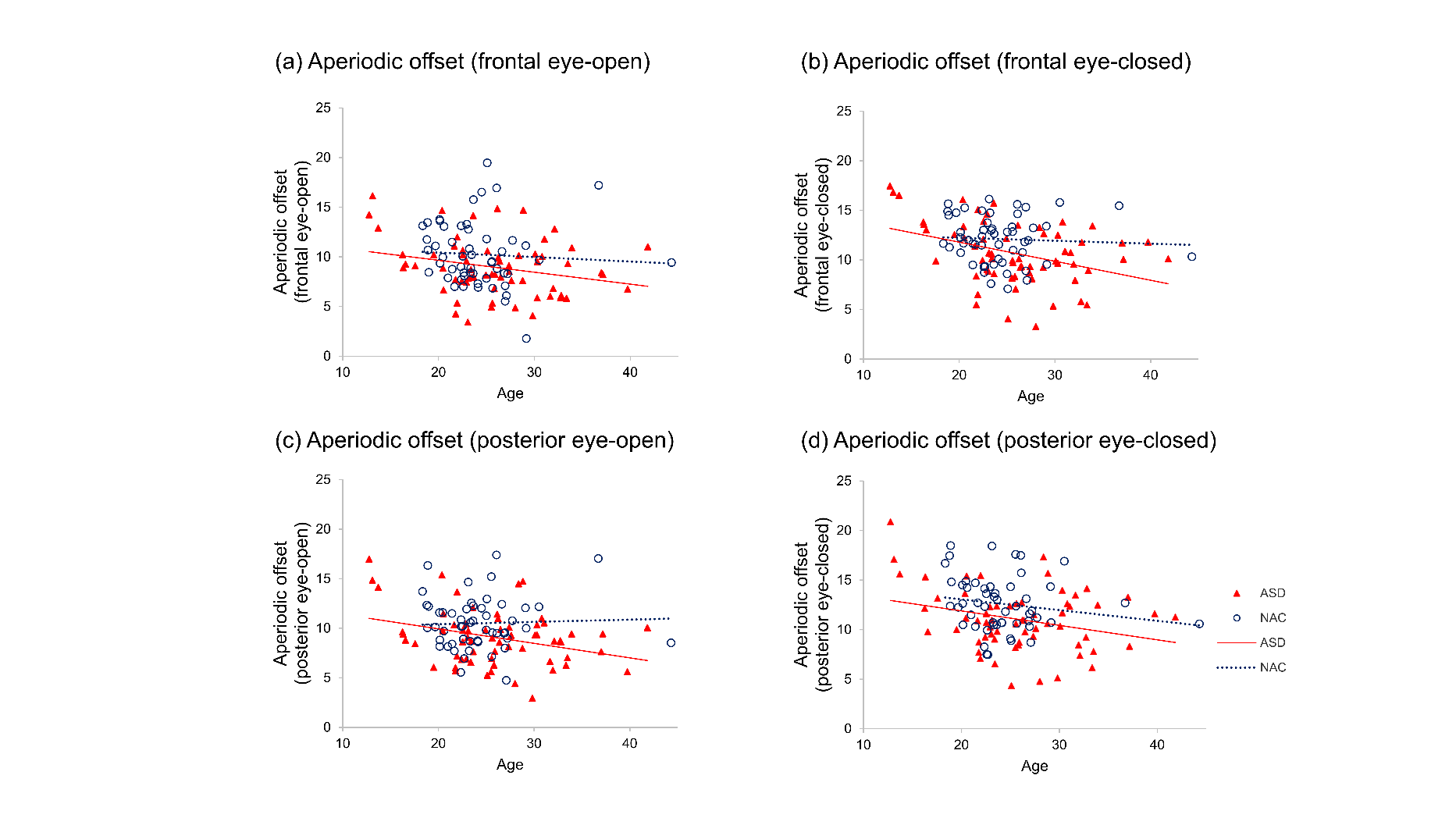


**Supplementary Figure S3.** Age-related changes of aperiodic offset


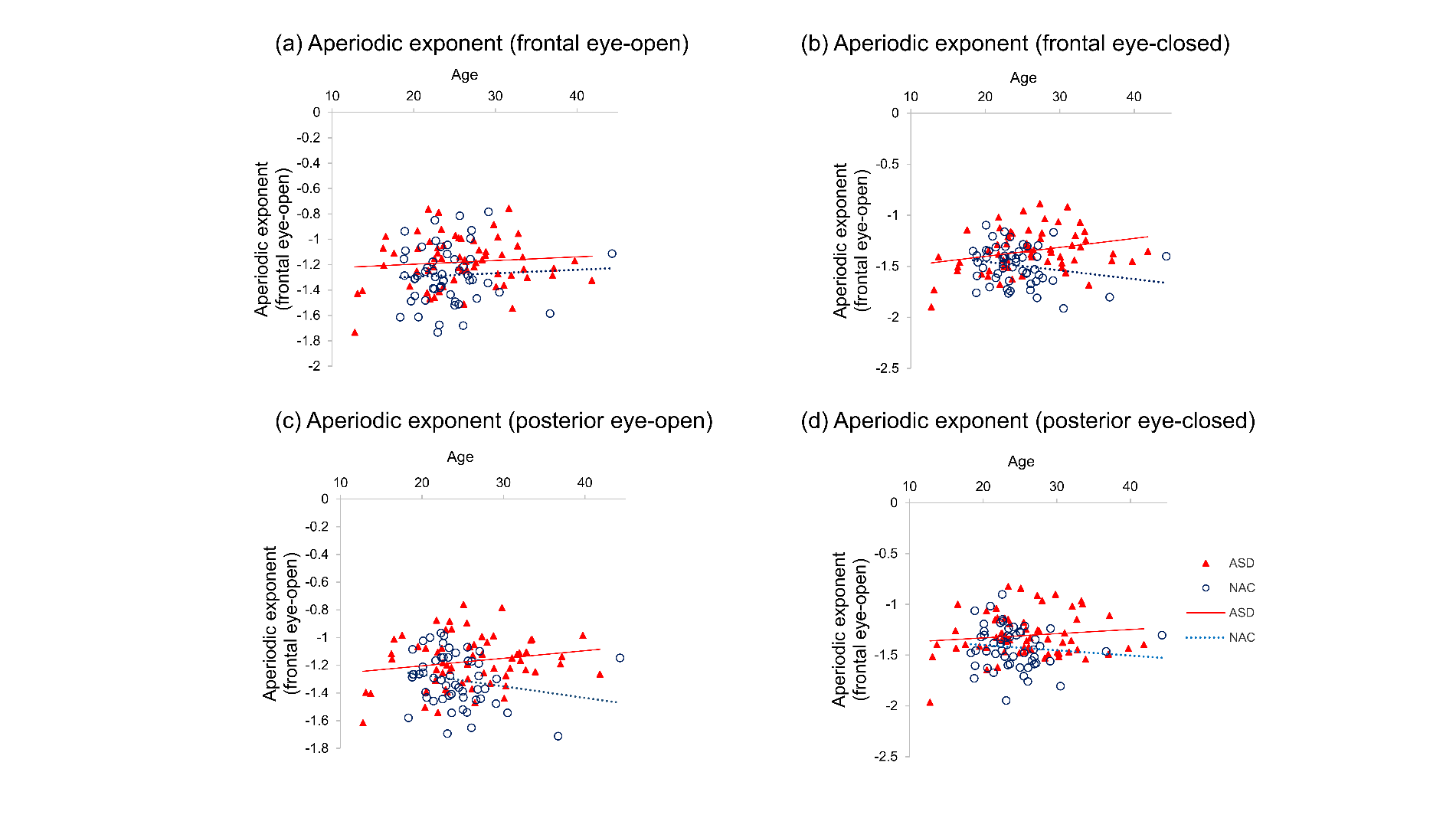

Supplement: Supplementary file 1 — Table S1. History of psychiatric comorbidities in autistic participants according to medical chart records. Table S2. Current or history of medications in autistic participants (all medications withheld on the day of electroencephalography [EEG]). Table S3. Group differences stratified by age 25, (A) ASD versus NAC in older subgroup (age > 25), (B) ASD versus NAC in younger subgroup (age ≤ 25). Table S4. Group‐by‐AQ/EQ interactions and Pearson's correlations between aperiodic components and AQ/EQ in ASD and in NAC. Figure S1. (A) Magnitude response of the zero‐phase FIR low‐pass filter (50 Hz cutoff, order = 132) showing a flat passband below 50 Hz. (B) Representative power spectrum displayed up to 70 Hz, illustrating preserved spectral features in the 35–45 Hz range. Figure S2. Age‐related changes of aperiodic exponent. Figure S3. Age‐related changes of aperiodic offset. [file PCN-80-423-s001.docx]
